# Characterization of the BspA and Pmp protein family of trichomonads

**DOI:** 10.1186/s13071-019-3660-z

**Published:** 2019-08-19

**Authors:** Maria R. Handrich, Sriram G. Garg, Ewen W. Sommerville, Robert P. Hirt, Sven B. Gould

**Affiliations:** 1 Institute for Molecular Evolution, Heinrich-Heine-University, Universitätsstraße 1, 40225 Düsseldorf, Germany; 2 Institute for Cell and Molecular Biosciences, Newcastle University, Newcastle upon Tyne, NE2 4HH UK

**Keywords:** *Trichomonas*, Adhesion, Infection, Pmp, BspA

## Abstract

**Background:**

*Trichomonas vaginalis* is a human-infecting trichomonad and as such the best studied and the only for which the full genome sequence is available considering its parasitic lifestyle, *T. vaginalis* encodes an unusually high number of proteins. Many gene families are massively expanded and some genes are speculated to have been acquired from prokaryotic sources. Among the latter are two gene families that harbour domains which share similarity with proteins of Bacteroidales/Spirochaetales and Chlamydiales: the BspA and the Pmp proteins, respectively.

**Results:**

We sequenced the transcriptomes of five trichomonad species and screened for the presence of BspA and Pmp domain-containing proteins and characterized individual candidate proteins from both families in *T. vaginalis*. Here, we demonstrate that (i) BspA and Pmp domain-containing proteins are universal to trichomonads, but specifically expanded in *T. vaginalis*; (ii) in line with a concurrent expansion of the endocytic machinery, there is a high number of BspA and Pmp proteins which carry C-terminal endocytic motifs; and (iii) both families traffic through the ER and have the ability to increase adhesion performance in a non-virulent *T. vaginalis* strain and *Tetratrichomonas gallinarum* by a so far unknown mechanism.

**Conclusions:**

Our results initiate the functional characterization of these two broadly distributed protein families and help to better understand the origin and evolution of BspA and Pmp domains in trichomonads.

**Electronic supplementary material:**

The online version of this article (10.1186/s13071-019-3660-z) contains supplementary material, which is available to authorized users.

## Background

Trichomonads constitute a large group of anaerobic parasites and commensals, most individual species of which have evolved host and tissue specificity. By far the best studied trichomonad is *Trichomonas vaginalis*, which thrives in the human urogenital tract [1, 2] and infects close to 300 million people annually [3, 4]. Symptomatic infection is significantly more frequent in females than in males, but only a minority lead to a fully-developed trichomoniasis [5, 6]. Given that most *T. vaginalis* infections remain unnoticed, this poses a problem since asymptomatic infections can still elevate the risk of developing cancer, facilitate the acquisition and transmission of HIV and other viruses, and are associated with a number of adverse pregnancy outcomes [5–8]. Treatments with a 5-nitroimidazole-based derivate are quite effective [9, 10], although about 10% of *Trichomonas* strains diagnosed display some tolerance or even resistance towards metronidazole-based drugs [10].

The pathogenicity of *Trichomonas* involves a variety of interactions with the host cell, e.g. the secretion of cysteine proteases that degrade the extracellular matrix and other substrates [11, 12], the secretion of exosomes that can fuse with human cells to deliver their content [13], and cell morphogenesis for adhesion [14–16]. Once amoeboid, *T. vaginalis* scavenges host cell substrate, likely through a mechanism similar to the trogocytosis of *Entamoeba histolytica* [17]. Another critical component is the endocytic uptake of food particles. During this process, extracellular material is recognized at the plasma membrane by specific receptors and usually placed into clathrin-coated vesicles by the assistance of the AP2 adaptor complex [18]. This process is further mediated by surface proteins, which carry specific sorting signals located at the cytosolic end of the proteins and which are recognized by complexes of the endocytic machinery leading to the internalization of these proteins [19]. It has been speculated that pathogenicity-relevant gene families, such as cysteine proteases and proteins of the Rab family of small GTPases, are specifically expanded in *T. vaginalis* [20]. These gene families appear selectively, and to a degree jointly, expressed upon different environmental stimuli [21].

The genome of *T. vaginalis* (strain G3) still remains the largest protist genome sequenced to date [20, 22]. This contradicts the usual trend, as parasite genomes tend to shrink as a consequence of evolutionary reduction [23]. The *T. vaginalis* genome is about seven times the size of the *Plasmodium falciparum* genome, more than 60 times that of *Encephalitozoon cuniculi* [22, 24], and substantially larger than that of other extracellular, excavate parasites such as *Giardia lamblia* (about 14 times) and *Trypanosoma brucei* (about 6 times) [25]. The current *T. vaginalis* genome sequence data are made of a loose collection of around 17,000 individual scaffolds, whose assembly is hindered by the presence of repetitive sequences that represent over 60% of the entire genome [20, 26]. Among the expanded gene families are some that encode surface proteins thought to mediate adhesion to host cells and other mucosal commensals of the local microbiota [20, 27–29].

The interaction of parasite surface molecules with the host cell surface is barely understood, although known to be crucial for infection [30, 31]. To date, the only human binding partner identified for *T. vaginalis* is galectin-1 that is bound by the parasite’s lipophosphoglycan coat [32]; even this single known interaction has been partly challenged [33]. Early screening of the genome for potential surface proteins unearthed several candidate families [27]. Subsequent proteomic analyses of the *T. vaginalis* surface identified about 140 membrane-bound surface proteins, including several members of the BspA-(Bacteroides surface protein A) and Pmp-(polymorphic membrane protein) family [34].

Both BspAs and Pmps are surface adhesion proteins of Bacteroidales/Spirochaetales and Chlamydiales, respectively. BspA proteins are leucine-rich repeat (LRR)-containing proteins characterized by a 23 amino acid long repetitive motif (called *Tp*LRR) that is typically found in the N-terminal region [35–37]. These surface proteins mediate host-pathogen interactions and promote cell aggregations [36, 38–40]. Moreover, BspA-deficient mutants of *Tannerella forsythia* are less likely to induce alveolar bone loss in mice [39], pointing to a pivotal role in virulence. Pmps are chlamydial surface proteins that mediate the initial binding of the obligate intracellular pathogen and eventual invasion into the host cell [41, 42]. Two consecutive tetra-peptide motifs, FxxN and GGA(I/L/V), are found in a repetitive manner in the N-terminal region of Pmps [43]. At least two copies of these motifs are required to mediate adhesion [41] and antibodies binding these N-terminal repeat motifs reduce the ability of Chlamydiae to infect by up to 95% [44].

BspAs have been found in other eukaryotic pathogens such as *Entamoeba*, where they were found to be involved in chemotaxis towards a tumor necrosis factor [45]. It is thought that genes encoding BspA and Pmp proteins were introduced into the genomes of eukaryotic pathogens through horizontal gene transfer (HGT) events [15, 46]. *Trichomonas vaginalis* G3 encodes 911 BspA-like and 48 Pmp-like proteins [15, 47]; expression evidence exists for more than half of them [21]. A few have been identified in surface proteomes (only about 1% of the encoded BspA proteins for instance [34]) but no dedicated functional analysis of either the *Tv*BspA or *Tv*Pmp proteins has been carried out. With the recent exception of *Dientamoeba fragilis* [48], the presence and diversity of BspA-like and Pmp-like genes among other trichomonad parasites remains unknown.

Here, we performed RNA-Seq on five trichomonads with a broad phylogenetic distribution. They include *Trichomitus batrachorum* (that infects the amphibian intestine), *Tetratrichomonas gallinarum* and *Trichomonas gallinae* (both parasites of various bird species), and *Pentatrichomonas hominis* and *Trichomonas tenax* (commensal species of the human intestine and oral cavity, respectively). We compared their expression data with that available for *T. vaginalis* to screen in particular for the presence of BspA and Pmp protein-encoding transcripts and to unravel their evolutionary trajectory in trichomonads. Expression of *T. vaginalis* candidate genes encoding BspA and Pmp proteins in a low-adhesive *T. vaginalis* strain and the galliform and anseriform bird-infecting *Tetratrichomonas gallinarum*, increases the ability of the parasites to adhere to a monolayer of vaginal epithelial cells by an uncharacterized mechanism. The unique expansion, particularly striking for the BspA family in *T. vaginalis*, underscores their importance regarding human-specific pathogenicity. Their diversity, for instance the partial absence of membrane-spanning regions and secretory signals in general, might suggest either unknown means of cell surface anchoring [47], divergent version of signal peptides not recognized *in silico* or functions other than surface-associated adhesion in trichomonad species.

## Methods

### Culturing

*Trichomonas vaginalis* strains T1 (kindly provided by Professor J. Tachezy, Charles University of Prague) and FMV1 (kindly provided by Professor M. Benchimol, University Santa Ursula) were cultured in tryptone-yeast extract maltose medium {2.22% (w/v) tryptose, 1.11% (w/v) yeast extract, 15 mM maltose, 9.16 mM l-cysteine, 1.25 mM l(+)ascorbic acid, 0.77 mM KH_2_PO_4_, 3.86 mM K_2_HPO_4_, 10% (v/v) horse serum, 0.71% (v/v) iron solution [= 25.5 mM Fe(NH_4_)_2_(SO_4_) × 6H_2_O, 4.5 mM (w/v) 5-sulfosalicylacid]} at 37 °C [49].

Vaginal epithelial cells (VECs, strain MS-74; [50]) were cultivated in 45% DMEM (#31885, Invitrogen, Carlsberg, USA), 45% keratinocyte-SFM (#37010022, Invitrogen) and 10% fetal calf serum (FCS) in standard cell culture flasks (75 cm^2^) at 37 °C and 5% CO_2_ in a Galaxy 48R (Eppendorf, Hamburg, Germany). For culture maintenance, cells were washed twice with Dulbecco’s PBS (#H15-001, PAA Laboratories, Pashing, Austria), digested with trypsin (#25300-054, Invitrogen) for 5 min and then inactivated with FCS. Cells were then pelletized at 755× *g* for 10 min, resuspended in fresh media and split 1:10 into new flasks and media. Finally, a penicillin/streptomycin mix was added to a final concentration of 100 µg/ml to prevent bacterial contamination.

### Transcriptomes of trichomonadids

RNA-Seq reads were obtained using Illumina sequencing based on *Pentatrichomonas hominis* PhGII (GenBank: SRX2052873), *Tetratrichomonas gallinarum* M3 (GenBank: SRA318841), *Trichomitus batrachorum* BUB GenBank: SRX2052874), *Trichomonas gallinae* GCB (GenBank: SRX2052872) and *Trichomonas tenax* HS-4 (GenBank: SRX2052871). RNA was isolated as previously described for *T. vaginalis* [51]. A quality-filtering step was applied to the reads so that the first nine nucleotide (nt) positions were rejected according to a FastQC analysis that showed low quality for the first 9 base calls. Subsequently, only reads with a minimum of 25 nt were retained. In addition, all reads containing 25% of low-quality bases (25% of all bases with values ≤ Q15) identified by a self-written Perl script were also rejected. The reads were assembled *via* Trinity assembler (v.20131110) [52]. From all assembled contigs only the longest isoform of a candidate was selected. Open reading frames (ORFs) were identified and translated into the corresponding amino acid (aa) sequences by *getorf* from EMBOSS v.6.6.0 [53] and a self-written Perl script was used to select only the longest ORF per candidate. To define an ORF, only stop codons were considered (option-find 0). Furthermore, only sequences with a minimum of 100 aa as a minimum for protein identification were used. For those sequences the best matches with *T. vaginalis* annotated genes were determined by using BLAST v.2.2.28 [54] in combination with the database TrichDB v.1.3 [55] based on an e-value cut-off at ≤ 1e^−10^.

### Endocytic motif search

Putative Pmp and BspA protein sequences were first analyzed for the presence of a transmembrane domain (TMHMM v.2.0) and only those with a minimum of one TMD were used for further examination. These sequences were then screened for the presence of endocytic motifs within the cytoplasmic tails using a custom perl script. The following search patterns were used: DxF (“D[A-Z]F”), FxDxF (“F[A-Z]D[A-Z]F”), WVxF (“WV[A-Z]F”), LLNLD (“LLNLD”), [DE]xxxL[LI] (“[DE][A-Z][A-Z][A-Z]L[LI]”), NPx[YF] (“NP[A-Z][YF]”), [FY]NPx[YF] (“[FY][A-Z]NP[A-Z][YF]”), YxxΦ (Y[A-Z][A-Z][FMVIL]”), DLYYDPM (“DLYYDPM”).

### Gene cloning and homologous expression of *T. vaginalis*

Candidate genes TVAG_166850, TVAG_183790, TVAG_140850, TVAG_212570 and TVAG_240680 were amplified using a proof-reading polymerase (Phusion High-Fidelity DNA Polymerase, #M0530S, NEB, Ipswich, USA) and cloned into *Trichomonas* expression vectors, which are all based on pTagvag2 (kindly provided by J. Tachezy and P. Dolezal, Charles University of Prague, Czech Republic) using the SCS-Promotor (TVAG_047890) for gene expression. Thirty micrograms of plasmid DNA were used for transfection of 2.5 × 10^8^ *T. vaginalis* cells using standard electroporation [56]. After 4 h of recovery, neomycine (G418) was added to a final concentration of 100 µg/ml for selection of positive transfected *T. vaginalis* cells. The correct expression of the fusion constructs was verified by specific reverse-transcriptase PCRs (Additional file 1: Figure S1).

### Reverse-transcriptase PCR

For RNA isolation, 50 ml of a dense grown culture (approx. 1 × 10^7^ cells/ml) was treated with Trizol™ reagent (#15596018, Thermo Fisher Scientific, Schwerte, Germany) according to the manufacturer’s guidelines. Afterwards, 500 ng of RNA was applied for DNase treatment using DNase I, RNase free (#EN0525, Thermo Fisher Scientific) and cDNA was synthesized by an iScript™ Select cDNA Synthesis Kit (#170-8896, Bio-Rad, Hercules, USA). A PCR was performed using the Phusion^®^ High-Fidelity DNA Polymerase (#M0530S, NEB) and the corresponding protocol. To ensure the amplification of the HA fusion constructs only, in each reaction gene specific primer were mixed with HA specific primer.

### Immunofluorescence assays

For immunofluorescent labelling, 12 ml of a dense grown *T. vaginalis* culture (approx. 1 × 10^7^ cells/ml) without dead cells, which accumulate as a cell pellet at bottom of the tube were centrifuged for 5 min at 900× *g* and RT. Supernatant was discarded and cells were gently resuspended in 500 µl of fixation-buffer [either 4% paraformaldehyde (16%; #15710, EMS, Hatfield, USA) or 4% paraformaldehyde + 0.05% glutaraldehyde in pre-warmed culture medium] and incubated for 30 min at 37 °C followed by centrifugation at 900× *g* and RT for 5 min. Cells were gently washed in PBS and centrifuged again under the same conditions. Supernatant was discarded and cells were resuspended in 100–150 µl of PBS depending on size of the cell pellet. Subsequent steps were performed in a 6-well plate (#83.3925, Sarstedt, Nümbrecht, Germany). Cell suspension was spread on a Poly-l-lysine (#P4707, Sigma-Aldrich, St. Louis, USA) coated coverslide and incubated for 30 min. After incubation, the suspension was gently removed from the 6-well slot and cells were incubated for 20 min in permeabilization-buffer (0.1% TritonX-100 in PBS) at RT on a 2D shaker. Alternatively, a 10 min treatment with 0.1% NP-40 was performed or 10 µg/ml digitonin was used for solubilization either for 10 or 30 min. After permeabilization, cells were washed three times briefly in PBS followed by a blocking step for 60 min in blocking PBS (1% BSA, 0.25% gelatine, 0.05% Tween20 in PBS) at RT on a 2D shaker. Blocking was followed by a brief washing step and then incubation with the first antibody (monoclonal anti-HA, produced in mouse; #H9658, Sigma) at a concentration of 1:500 in blocking PBS for 1 h at RT followed by 4 °C overnight. Samples were washed three times for 5, 10 and 15 min each before incubation with secondary antibody 1:1000 (donkey anti-mouse IgG, Alexa Fluor 488, #A21202, Thermo Fisher Scientific) for 2 h at RT. After three washing steps (5, 10 and 15 min) samples were mounted with Fluorshield containing DAPI (#F6057, Sigma). Samples were stored at 4 °C until imaging.

For immunofluorescence assays in the presence of vaginal epithelial cells (VECs MS-74), one day prior to the experiment, 500 µl of VECs were placed onto each slot of a 4-well CultureSlide (#354114, BD Falcon, Schaffhausen, Switzerland) and incubated overnight at 37 °C and 5% CO_2_ in a Galaxy 48R (Eppendorf). The next day, 12 ml of a dense grown *T. vaginalis* culture (approx. 1 × 10^7^ cells/ml) were centrifuged at 900× *g* and RT for 5 min. The supernatant was discarded and the pellet was washed once with 500 µl of PBS. After a second centrifugation step the pellet was resuspended in 100–150 µl of PBS and the complete cell suspension was placed into one slot of the culture slide with pre-incubated VECs. After 30 min incubation at 37 °C and 5% CO_2_, fixation buffer [4% paraformaldehyde (16%) in PBS] was added and cells were again incubated for 30 min at 37 °C and 5% CO_2_. Permeabilization as well as antibody treatment were performed according to the above protocol. For lysosome co-localization studies, cells were first incubated with 130 nM LysoTracker™ Red DND-99 (#L7528, Thermo Fisher Scientific) diluted in pre-warmed culture medium for 2 h at 37 °C.

### Live cell imaging

For the detection of the GFP tagged fusion proteins, 2 ml of a dense grown culture (approx. 1 × 10^7^ cells/ml) was centrifuged at 900× *g* for 5 min and the pellet was carefully resuspended in 150 µl of PBS and placed on a Poly-l-lysine (#P4707, Sigma-Aldrich) coated coverslide and incubated at 37 °C and 5% CO_2_ in a Galaxy 48R (Eppendorf). After 1 h of incubation, the cells were washed once with 1 ml PBS and samples were mounted with Fluorshield containing DAPI (#F6057, Sigma) and immediately used for microscopy.

### Adhesion assays

Adhesion assays were performed three times in triplicates for each candidate. One day prior to the adhesion assay, 1.5 × 10^5^ vaginal epithelial cells (VECs MS-74) were placed into each slot of a 4-well CultureSlide (#354114, BD Falcon) and filled up to a final volume of 1 ml with culture medium [45% DMEM (#31885, Invitrogen), 45% keratinocyte-SFM (#37010022, Invitrogen) and 10% fetal calf serum (FCS)]. Vaginal epithelial cells were incubated overnight at 37 °C and 5% CO_2_ in a Galaxy 48R (Eppendorf). Preliminary to the assay, *T. vaginalis* cells of a dense grown culture (approx. 1 × 10^7^ cells/ml) were incubated with 10 µM CellTracker Green CMFDA Dye (#C7025, Invitrogen) for 30 min at 37 °C and 5% CO_2_ in *Trichomonas* culture medium [2.22% (w/v) tryptose, 1.11% (w/v) yeast extract, 15 mM maltose, 9.16 mM l-cysteine, 1.25 mM l(+)ascorbic acid, 0.77 mM KH_2_PO_4_, 3.86 mM K_2_HPO_4_, 10% (v/v) horse serum, 0.71% (v/v) iron solution [25.5 mM Fe(NH_4_)_2_(SO_4_) × 6H_2_O, 4.5 mM 5-sulfosalicylacid]. The culture was spun down at 500× *g* for 5 min at RT and washed two times with PBS. *Trichomonas vaginalis* cells were counted (TC20 Automated Cell Counter, BioRad) and for each assay infection was initiated with 5 × 10^4^ *T. vaginalis* cells in 500 µl of PBS and incubated at 37 °C and 5% CO_2_ for 30 minutes. After incubation free swimming cells were washed off with PBS and adherent cells were fixed in PBS containing 1% paraformaldehyde (#15710 EMS) for 30 min at 37 °C and 5% CO_2_. Observation at a microscope was performed at 10× magnification and for each assay 12 closely spaced pictures were taken. Quantitative analysis of the adherent cells was performed with ImageJ v.1.48.

## Results

### RNA-Seq on five trichomonads

We first generated RNA-Seq data for five trichomonad species that infect a variety of different hosts (Table 1), namely *Pentatrichomonas hominis*, *Tetratrichomonas gallinarum*, *Trichomitus batrachorum*, *Trichomonas gallinae* and *Trichomonas tenax*. For functional classification and comparison to the human parasite, the assembled transcriptomes (i.e. the assembled open reading frames, ORFs) were blasted against *T. vaginalis*, for which comparable transcriptome data were generated previously [21] and a sequenced genome is available [20]. Both the number of assembled ORFs among the different analyzed species and the number of ORFs with homologs in *T. vaginalis* differed (Table 1). With 57% overlap, the lowest number of homologs in *T. vaginalis* were found for *Tri. batrachorum* and with 89% overlap the highest number in both *T. gallinae* and *T. tenax*, which is in accordance with their phylogenetic relationships [57, 58]. Considering the predicted genome sizes [22], there is no apparent correlation between these and the number of expressed genes. The data add additional credit to the dynamic and expanded nature of the genomes this protist group is known for [21, 59].

**Table 1:** RNA-Seq details of the trichomonads sequenced

Species	Strain	Host	ORF screen^a^	Homologs in *T. vaginalis*	%^b^
*T. gallinae*	GCB	Parasite: digestive tract of columbiform and falconiform birds	21,996	1953	89
*T. tenax*	HS-4	Commensal: oral cavity of humans, dogs and cats	24,071	2143	89
*Tet. gallinarum*	M3	Parasite: intestine of galliform and anseriform birds	37,740	2613	69
*Tri. batrachorum*	BUB	Parasite: intestine of amphibians	34,415	1950	57
*P. hominis*	PhGII	Commensal: intestine of mammals including humans	46,458	3234	70

^a^ORF identified among distinct contigs derived from RNA-Seq reads

^b^Percentage of ORFs with homologues in *T. vaginalis*

**Fig. 1:**
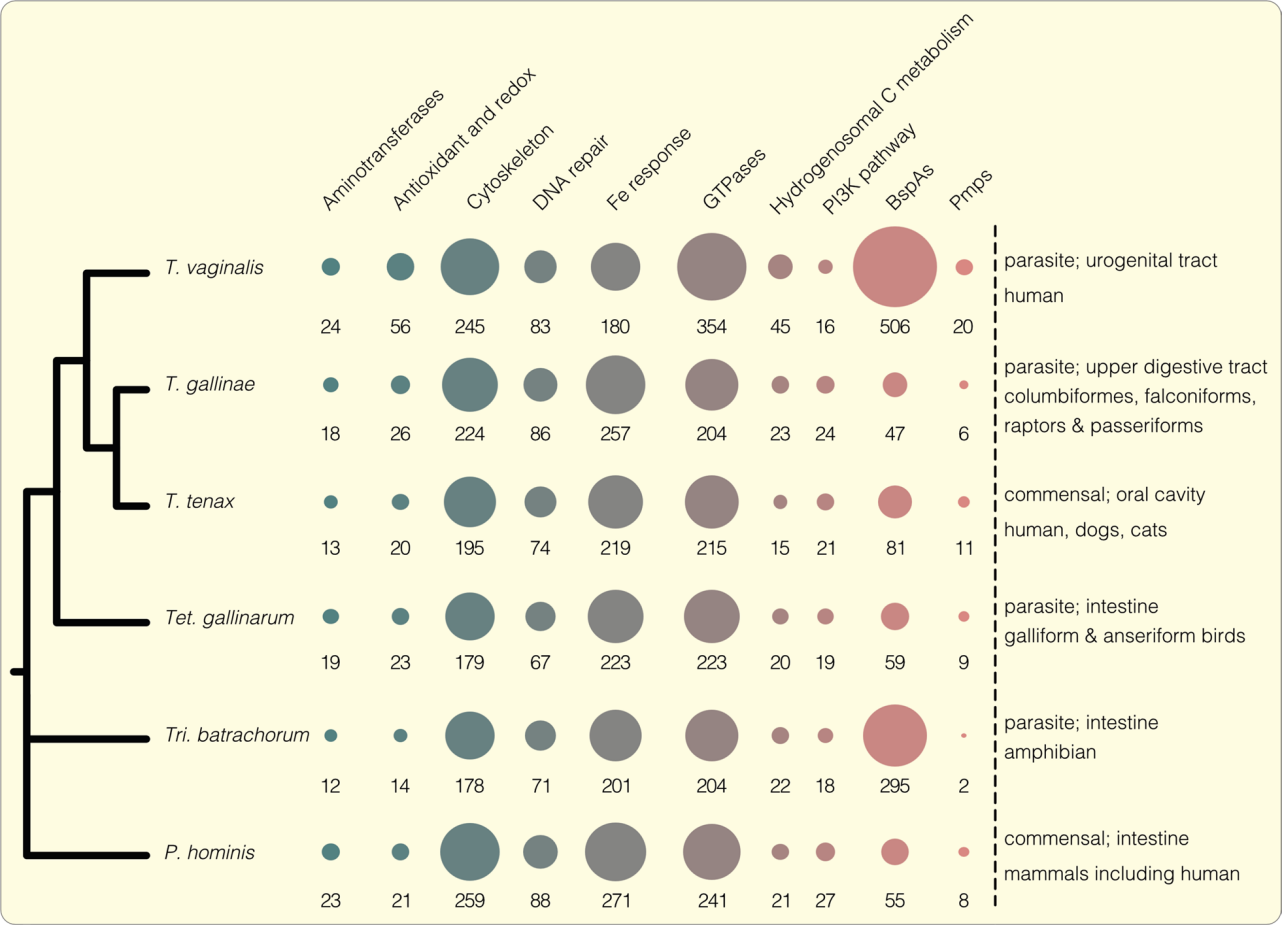
Comparative analysis of expression levels of specific gene families among trichomonads. For our analysis we selected different trichomonad species, both commensal and parasitic ones, with a high diversity of host cells. The amount of expressed transcripts belonging to different housekeeping gene families or proteins possessing BspA and Pmp specific domains are represented as bubbles. On average the size of the transcribed gene families in *T. vaginalis* are doubled compared to the other trichomonads. In particular, the expression of genes belonging to the BspA and Pmp protein families are almost exclusively expanded in *T. vaginalis*

Comparing the size of gene families that are expressed in *T. vaginalis* with that of the other trichomonads demonstrates that the human pathogen does differ (Fig. 1). On average, the screened transcribed gene families of *T. vaginalis* were about twice the size in comparison to those of the other trichomonads. In particular, genes encoding either for proteins of the BspA or Pmp family stood out. Notably, a similar expansion of expression levels was observed for genes encoding proteins of the BspA family in *Tri. batrachorum* (Fig. 1), a parasite of the amphibian intestine. Comparing the estimated genome sizes of trichomonads [22] and the assembled transcriptomes, no direct correlation can be made. Most importantly, it is evident that variants of both the BspA and Pmp gene family, suspected to be associated with increasing host cell adhesion, are expressed by all of the trichomonads that we analyzed. The transcription levels of both families, however, are noticeably expanded only in *T. vaginalis*.

### The BspA and Pmp protein family of trichomonads

**Fig. 2:**
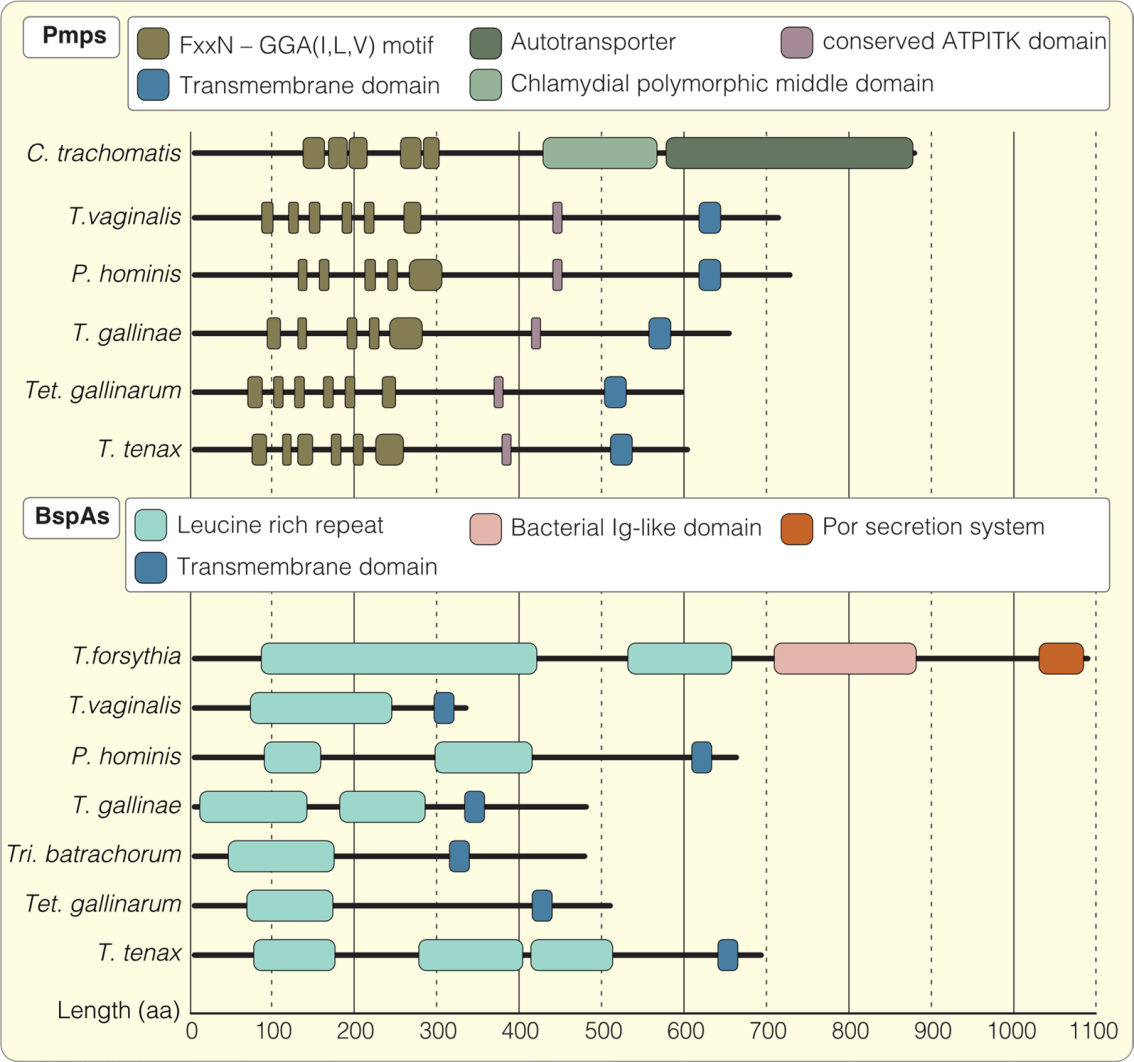
Structural comparisons of the Pmp and BspA protein family in different trichomonads. Schematic illustration of selected Pmp structures in different trichomonadida together with the prokaryotic model *Chlamydia trachomatis* (accession numbers: *C. trachomatis*, gi 34539119; *T. vaginalis*, TVAG_249300; *P. hominis*, PEHa011017; *T. gallinae*, TEGb004672; *Tet. gallinarum*, TRGa004464; *T. tenax*, TRTa003481). The Pmp family is characterized by multiple repeats of the FxxN and GGA[I/L/V] motifs located in the N-terminal region but compared to *C. trachomatis* the trichomonad proteins miss the C-terminal polymorphic middle and autotransporter domain. Instead they possess a conserved ATPITK motif as well as a transmembrane domain at their C-terminus. Selected members the BspA family of different trichomonadida compared to one of *Tannerella forsythia* (accession numbers: *T. forsythia*, gi 3005673; *T. vaginalis*, TVAG_240680; *P. hominis*, PEHa029834; *T. gallinae*, TEGb004448; *Tri. batrachorum*, TRBa028008; *Tet. gallinarum*, TRGa003876; *T. tenax*, TRTa008806). The BspA protein family is unified by several N-terminal copies of leucine-rich-repeat elements. In *T. forsythia* the BspA additionally possess a bacterial Ig-like domain (Big-domain) as well as a por secretion system both localized towards the C-terminus. In the trichomonads, those are replaced by a transmembrane domain in a proportion of BspA-like proteins

The increased expression of both the BspA and Pmp protein family in the human pathogen *T. vaginalis* raises the question about their distinct roles during infection, or more precisely whether those proteins are implicated in directly mediating adhesion to human host tissue as has been previously speculated [15, 27]. Structural comparison of the two protein families revealed several similarities among the trichomonads, which are consistent with the built up found for prokaryotic homologs (Fig. 2). Similar to their prokaryotic counterparts, the Pmp-specific repeat motifs FxxN and GGA[I/L/V] and the leucine-rich-repeats of the BspA family are present throughout the main parts of the respective proteins towards their N-termini [43, 60]. Besides these conserved regions, both families have undergone similar modification towards their C-terminus, in particular the substitution of prokaryote-specific elements (such as the por secretion system or the autotransporter domain) [61, 62] with a single membrane spanning domain close to the C-terminus.

**Fig. 3:**
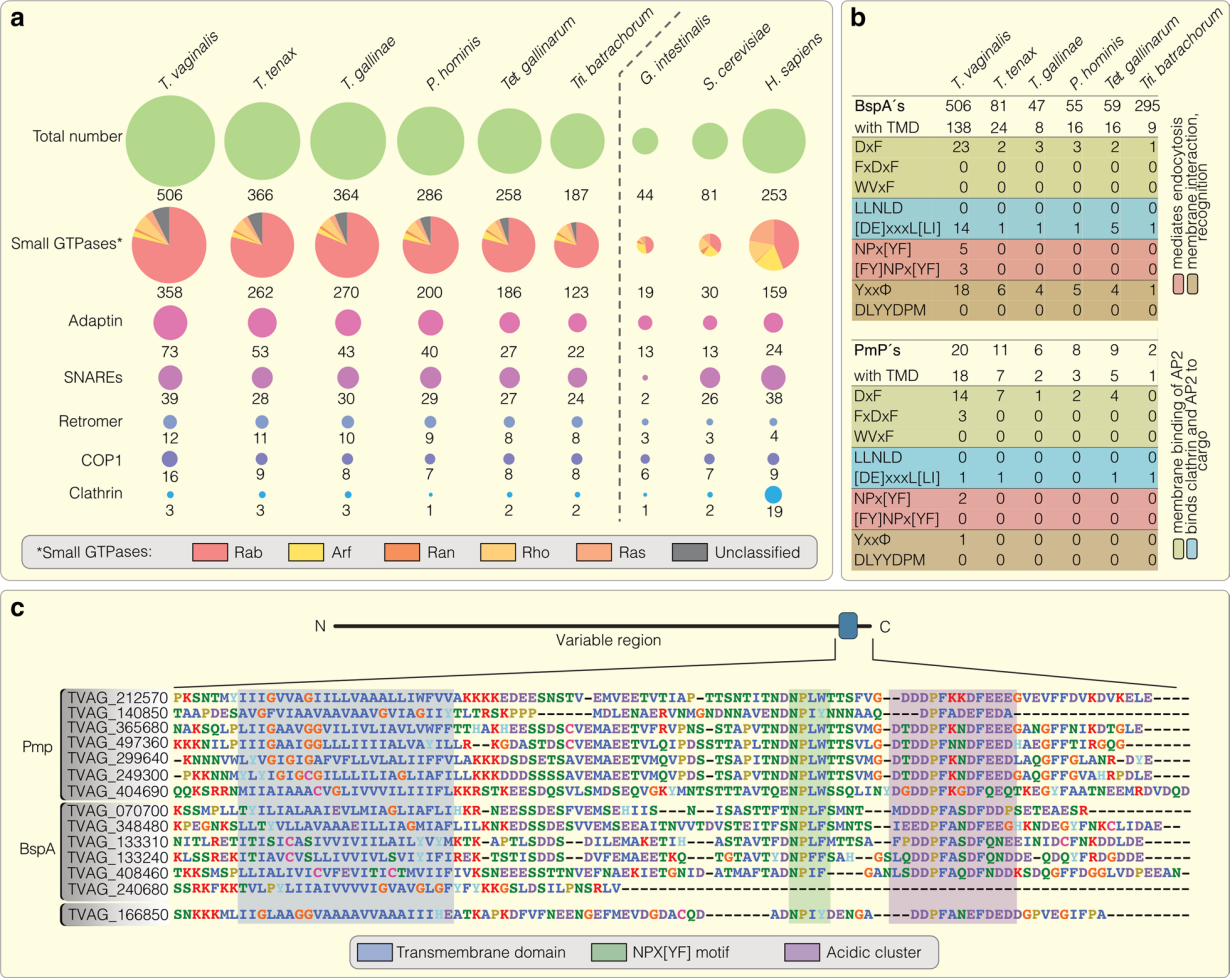
Increased expression of endocytosis-related protein families and analysis of specific endocytic motifs within the Pmp and BspA protein family. **a** Expression levels of several protein families involved in endocytosis are represented as bubbles and compared among the trichomonads. Although *T. vaginalis* again shows the highest numbers, all gene families are expanded in the trichomonads compared to *Giardia intestinalis* another anaerobic human parasite. In particular, the Rab subfamily of the small GTPases displays a specific expansion in transcription among the trichomonadida as obvious by comparing those numbers to the human gene family. **b** The trichomonad Pmp and BspA families were screened for the presence of a transmembrane domain and further for specific endocytic motifs within their cytoplasmic tails. While 90% of the *T. vaginalis* Pmps carry a TMD it is just around a quarter for the BspAs. Notably, in *Tri. batrachorum*, which showed a similar expansion of this protein family, only around 3% exhibit a TMD. The fraction of proteins with motifs involved in endocytic processes is diverse. However, the *T. vaginalis* proteins possess an increased amount of specific motifs e.g. the NPx[YF]. **c** Sequence alignment of *T. vaginalis* Pmp and BspA proteins with focus on the C-terminal region. Together with the conserved transmembrane domain those proteins [with one exception (TVAG_240680)] also carry the *T. vaginalis* specific NPx[YWF] motif and an acidic cluster within their cytoplasmic tails

*Trichomonas vaginalis* is an extracellular parasite. During infection it faces host immune defense mechanisms and interacts with the urogenital microbiota, of which some is also phagocytosed [63, 64]. The abundance of BspA and Pmp proteins with endocytic motifs likely reflects a specific need and the question follows whether the endocytic machinery was expanded in the same way. We screened for proteins required for vesicle formation at the plasma membrane [65–67], vesicle fusion [68, 69] and proteins involved in intracellular trafficking [70, 71]. Indeed, in comparison to e.g. *Giardia intestinalis*, the expression levels of genes belonging to the protein families in question are expanded among all trichomonads and especially so in *T. vaginalis* (Fig. 3a). In particular, the Rab subfamily of small GTPases stood out: representing 75% of the GTPases in this single celled organism, the contribution of the Rab family is remarkably higher than in humans, a metazoan with tissue-specific expression where they comprise less than 50% [72]. These results also match former findings where an increased gene copy number of genes encoding for small GTPases and Rab proteins was observed in the *T. vaginalis* G3 strain [20]. Furthermore, this expansion is comparable to the *T. vaginalis* BspA and Pmp family, for which transcription is also specifically expanded. Hence, it seems likely that those proteins are also involved in the endocytic machinery.

The initial screen for proteins possessing a TMD generally resulted in comparable relative values. Only in *Tri. batrachorum*, which showed an increased expression of the BspA family similar to that of *T. vaginalis*, the amount of TMD containing proteins is considerably lower. Screening these proteins for motifs uncovered several that are recognized by the endocytic machinery (Fig. 3b), which are mostly located within the C-terminal tails. We found a DxF motif involved in membrane binding of the AP2 complex [19, 73] present in proteins of almost all trichomonads analyzed, while the NPx[YF] motif seems to be restricted to *T. vaginalis*. Sequence alignment of selected Pmps revealed a frequent motif modification, in particular a substitution of phenylalanine with tryptophan in *T. vaginalis* which does not necessarily render the motif dysfunctional. Similar functional substitutions have been observed in targeting motifs of secondary red algae [74]. Furthermore, a conserved acidic cluster in both protein families is evident (Fig. 3c), which represents another family of membrane-sorting signals [19, 75]. Nevertheless, there is a large domain diversity observed among both protein families and overall no generalized pattern is recognizable.

### Individual *T. vaginalis* BspA and Pmp proteins increase the adhesion performance

In order to investigate the influence of the Pmps and BspAs on adhesion, we selected candidate proteins and expressed them in the low-adhesive *T. vaginalis* T1 strain and the bird pathogen *Tetratrichomonas gallinarum*. We chose candidate proteins [BspA TVAG_240680 (36 kDa), Pmp TVAG_212570 (68 kDa) and Pmp TVAG_140850 (67 kDa)] that are generally expressed at higher levels or even upregulated upon exposure to host cells [21] and which displayed an increased abundance in highly adherent strains [34].

**Fig. 4:**
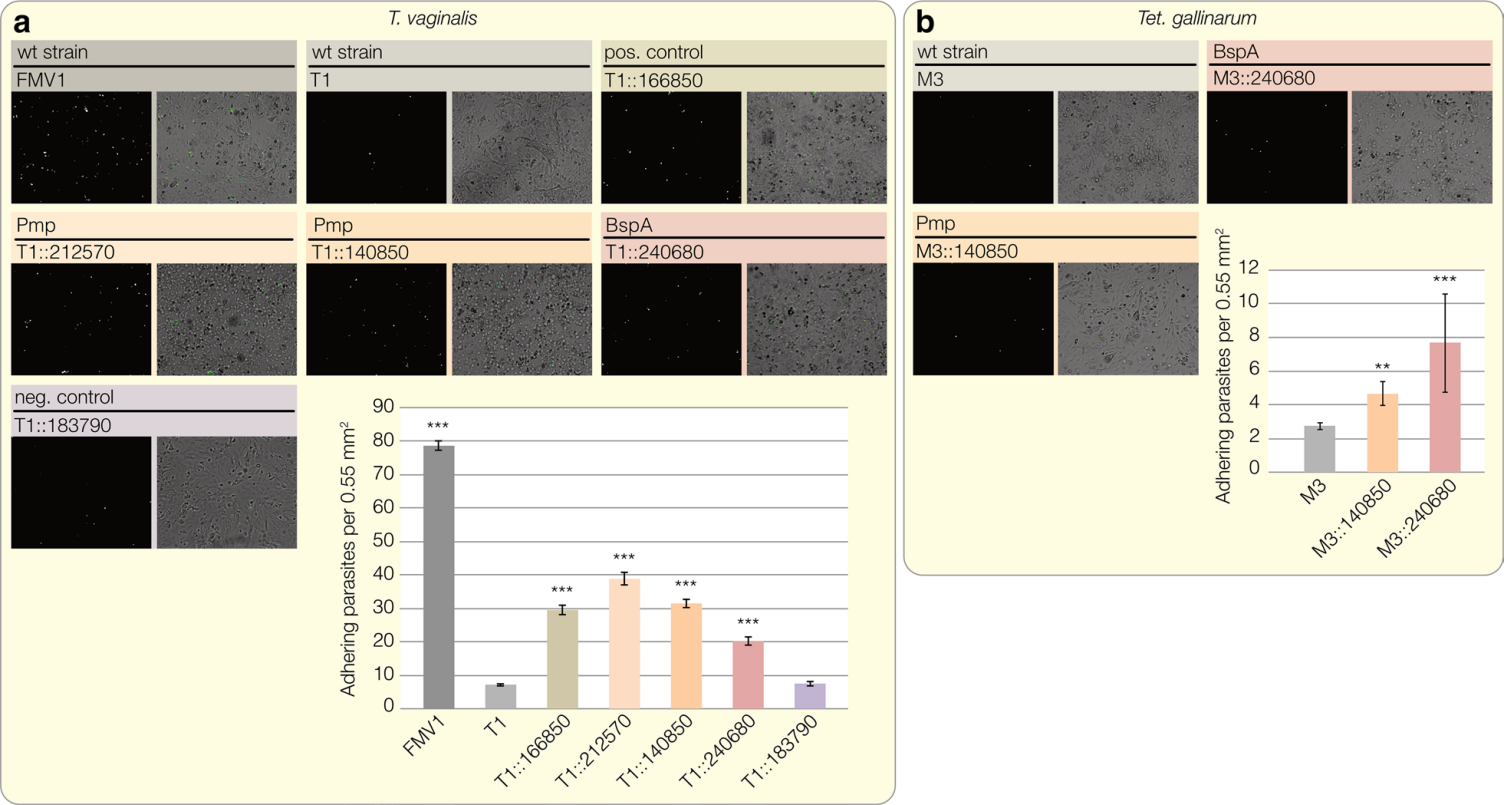
Adhesion assays for *T. vaginalis* and *Tet. gallinarum* on vaginal epithelial cells. **a** Overexpression of specific Pmp and BspA proteins increases the adherence of the low-infective T1 strain to vaginal epithelial cells (VECs). Example pictures for every assay are shown on the top where each white or green dot represents one adhering parasite. Compared to the T1 wt expression of the candidate Pmp (TVAG_212570 and TVAG_140850) and BspA (TVAG_240680) proteins significantly increased the number of attached *T. vaginalis* cells. The highly virulent FMV1 wt strain and malic enzyme (TVAG_183790) were used as a positive and negative control, respectively. Adhesion assays were performed nine times independently and for each candidate a total of 108 pictures were analyzed. T-tests were performed for the analysis of statistical significance compared to *T. vaginalis* wildtype strain T1 (***P < 0.0001, **P < 0.001, *P < 0.05). **b** The expression of TVAG_140850 (Pmp) and TVAG_240680 (BspA) also significantly increases the ability of the bird infecting pathogen *Tet. gallinarum* M3 wt to adhere to human host cells. Adhesion assay was performed three times independently and for each candidate a total of 36 pictures were analyzed. T-tests were performed for the analysis of statistical significance compared to *Tet. gallinarum* wildtype strain M3 (***P < 0.0001, **P < 0.001, *P < 0.05)

T1 cells expressing candidate proteins, as also shown by reverse-transcriptase PCR experiments (Additional file 1: Figure S1, were used to perform adhesion assays. All tested candidate proteins increase the adherence to VECs two to four-fold in comparison to the T1 wt strain (Fig. 4a). This is only half of the number of adhering cells counted for the virulent FMV1 strain, but still matches the results of the positive control [TVAG_166850 (83 kDa)] that is known to facilitate the adhesion of *T. vaginalis* [34]. The expression of malic enzyme (TVAG_183790), a protein of hydrogenosomal energy metabolism (and our negative control), did not lead to increased adherence. Furthermore, we expressed the BspA and one Pmp candidate protein in *Tet. gallinarum*, a parasite usually infecting the digestive tract of birds, and analyzed their influence on the binding to vaginal epithelial cells. Although the overall adherence was lower compared to *T. vaginalis* cultures expressing the same candidate proteins, both lead to a significant increase of adhering parasites. Compared to the M3 wildtype strain, the number of adhering cells was more than 1.5-fold increased in the case of the Pmp, and more than 4-fold higher for the *Tet. gallinarum* clone expressing the BspA protein (Fig. 4b).

### BspA and Pmp proteins localize predominantly to internal compartments, not the plasma membrane

**Fig. 5:**
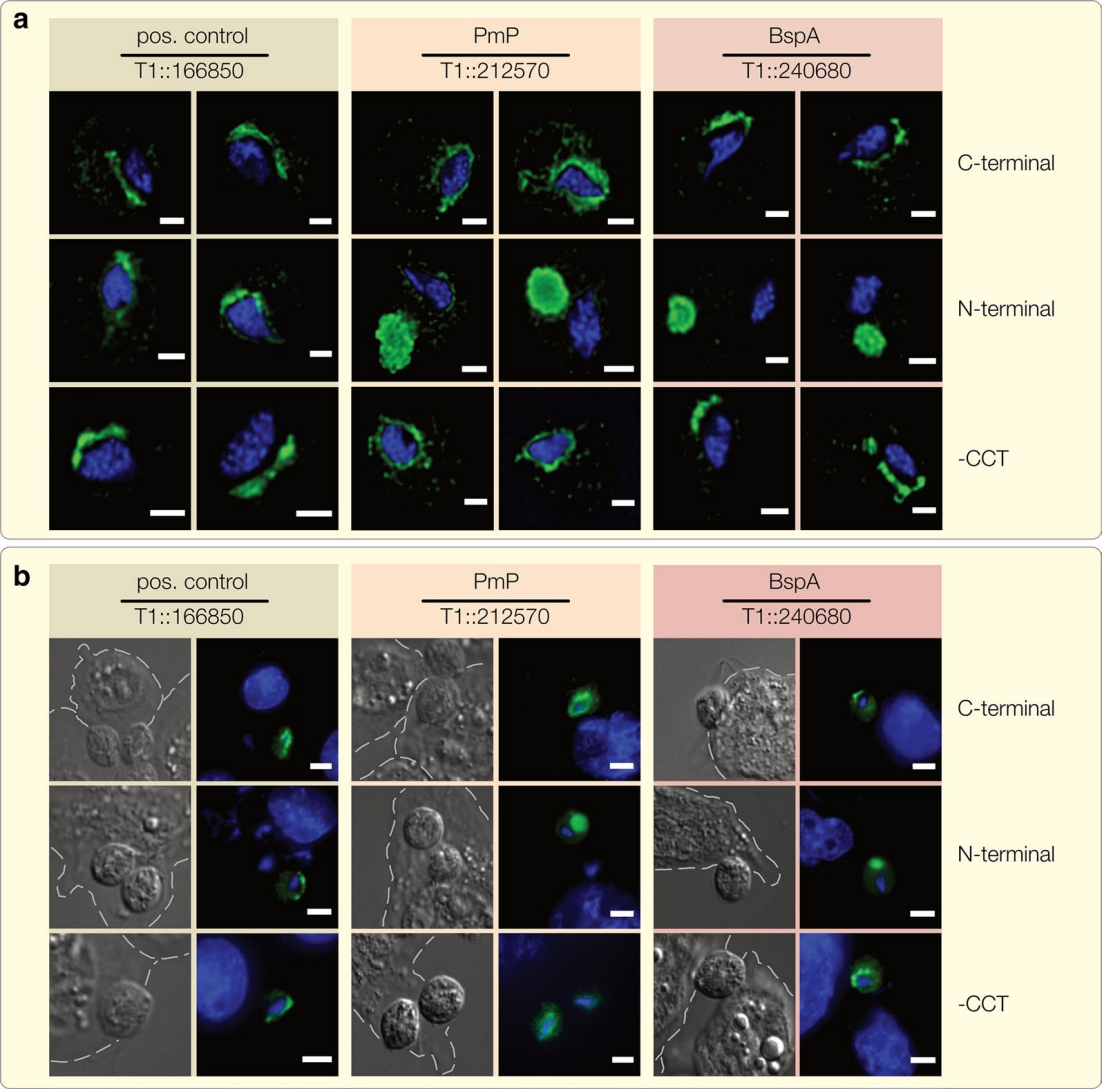
BspA and Pmp proteins localize predominantly to the ER and Golgi. The subcellular localizations of the positive control (TVAG_166850) and the candidate Pmp (TVAG_212570) and BspA (TVAG_240680) protein were analyzed using a specific antibody against the HA-tag. The protein localizations are shown (green) together with the nucleus (blue) which was stained using 4′,6-diamidin-2-phenylindol (DAPI). **a** The C-terminal tagged proteins which were used for adhesion assays constantly show a localization to the ER and Golgi apparatus. While shifting this tag position to the N-terminus has no influence on the positive control (which is still found to reside in the ER) it leads to a relocalization of both the Pmp and BspA to the lysosome. In contrast, removing the cytoplasmic tails (by replacing it with the HA-tag) did not lead to any localization shift compared to the C-terminally tagged proteins analyzed first. **b** We further checked if those localizations are influenced by the presence of host cells, but no change could be observed for *T. vaginalis* cells grown on VECs. White dashed lines highlight the periphery of the human cells. *Scale-bars*: **a**, 2 µm; **b**, 5µm

**Fig. 6:**
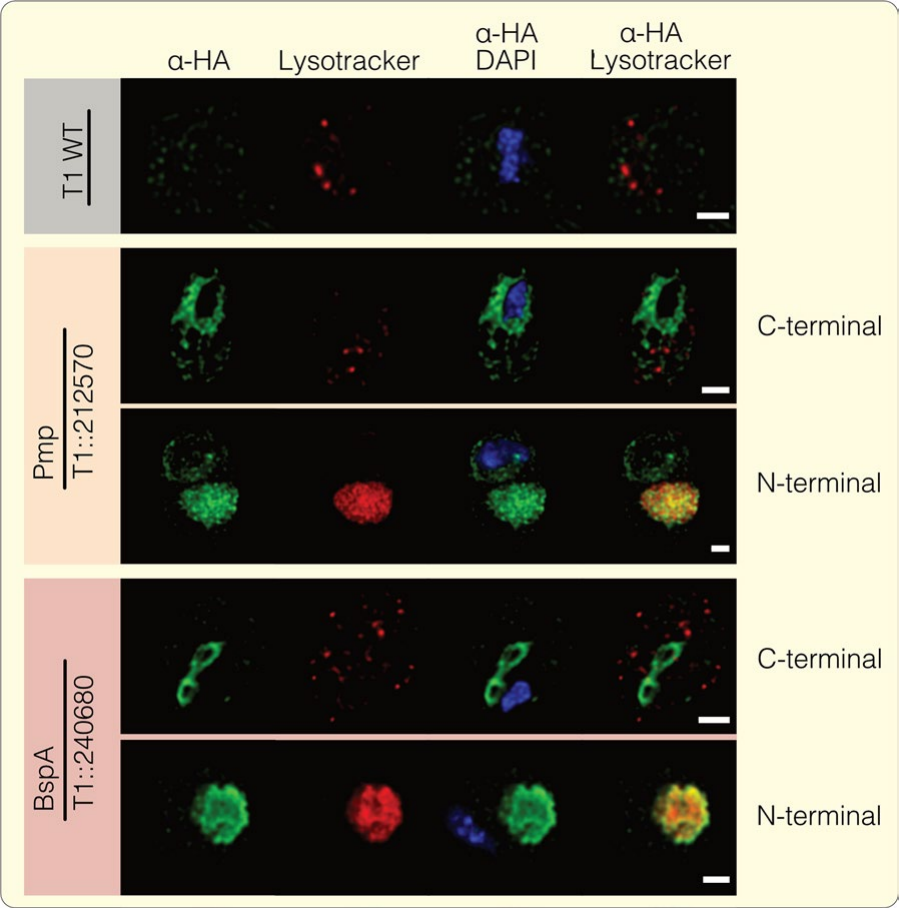
Lysosome localization of the N-terminal tagged Pmp and BspA protein. To verify the observed localizations of the N-terminal tagged Pmp and BspA protein, a co-localization with LysoTracker (red) was performed. The candidate proteins were detected using a specific α-HA antibody (green) while 4′,6-diamidin-2-phenylindol (DAPI) was used for nucleus staining (blue). In the T1 wt strain as well as the candidate proteins which carry the HA-tag at the C-terminus several small lysosomes were detected, equally distributed in the cytosol. Simultaneously the Pmp and BspA show circular structures around and near the nucleus, respectively. In contrast, one greatly enlarged lysosome could be detected in the N-terminally tagged cultures which clearly co-localizes with the HA-fusion constructs. *Scale-bar*: 2 µm

Next, we analyzed the subcellular localization of the candidate proteins used for the adhesion assays. The Pmp and BspA proteins, as well as the positive control (TVAG_166850), localize to structures that are reminscent of the endoplasmic reticulum (ER) and Golgi apparatus of the parasite (Fig. 5a) [76–78]. This was unexpected but observed in at least three independent experiments for each protein. To exclude the possibility that this localization was the result of C-terminal HA-tagging, which could interfere with the C-terminally localized endocytic motifs, we also tagged the proteins at their N-termini (Fig. 5a). Both N-terminally tagged constructs localize to a single spherical large lysosome mainly located sideward behind the nucleus, which is evident by the co-localization with LysoTracker (Fig. 6, Additional file 2: Figure S2). However, in cell lines expressing C-terminally tagged constructs, we observed many small lysosomes (Fig. 6), which corresponds to what is usually described for *T. vaginalis* [76, 79]. The cells in which the fusion proteins localize to the lysosomes, also perform poorly in terms of increasing adhesion performance (Additional file 3: Figure S3). In contrast, the substitution of the cytoplasmic tails that carry the motifs known to be recognized by the endosomal machinery through an HA-tag, did not alter ER/Golgi localization (Fig. 5a).

We also considered whether the exposure to host tissue could trigger a change in localization. However, the observed localization of the proteins for cells growing in the absence of VECs did not differ from those that were exposed and found attached to VECs (Fig. 5b). Similarly, we expressed two of our candidate proteins, the positive control and the BspA, in the highly virulent FMV1 wildtype strain in order to exclude the possibility that the missing ability to fully adhere has an influence on the protein localizations, yet no localization change was observed (Additional file 4: Figure S4). Using Triton X-100 alone can over-extract membrane proteins [80], but the use of NP-40 or treatments with the milder detergent digitonin led to the same results as when using Triton X-100 (Additional file 5: Figure S5). Finally, to circumvent the use of any detergent, we tagged the proteins with the green fluorescent protein for live imaging. While the fluorescence was low (GFP requires O_2_ to fluoresce, but *Trichomonas* is an anaerobic parasite), again only a localization to the ER and Golgi was evident (Additional file 6: Figure S6).

## Discussion

To our knowledge, the only trichomonad genome that has been sequenced to date is that of *T. vaginalis*, although estimations exist for several other trichomonads [22]. We observed that no clear correlation between the predicted genome sizes and number of expressed genes is evident. Until genome data for other trichomonad species becomes available, such numbers need to be treated with caution. We therefore compared only the numbers of expressed genes, which demonstrates that among the different trichomonads, *T. vaginalis* generally expresses the largest number of genes from each family and especially so regarding the BspA and Pmp families. These numbers highlight the importance of the two gene families and their universal presence among the trichomonads tells us something about their origin.

Previous analyses have suggested that the BspA and Pmp family were acquired through HGT from different prokaryotic sources [46, 81]. RNA-Seq data of all five trichomonads and *T. vaginalis* contained a substantial number of transcripts encoding proteins of the Pmp and BspA family. This argues for an ancient origin in trichomonads, which is supported by the additional presence of the BspA family in the tritrichomonad *Dientamoeba fragilis* [48]. One could argue for an ancient acquisition from mucosal-dwelling prokaryotic microbiota, although independent acquisitions through HGT cannot be ruled out. Still, the frequency of HGT in eukaryotes remains a controversial topic [82, 83]. If these proteins trace back to HGT and a prokaryotic source, it occurred before the diversification of parabasalia. Any phylogenetic analysis is impeded by the low sequence identity of eukaryotic BspAs and Pmps, which is furthermore restricted only to the repetitive motifs of the N-termini. Hence, if they are of prokaryotic origin, then early in their parabasalid evolution, the C-terminal prokaryotic domains were substituted with eukaryotic ones, which often contained a single TMD and endocytic motifs (Figs. 2, 3c). Especially in *T.* *vaginalis* and *Tri. batrachorum* not entire genes but only those sections useful for the eukaryotic parasite were retained and expanded.

Concomitant with the increased transcription of genes of the BspA and Pmp protein family and the presence of C-terminal motifs known to be recognized by the endocytic machinery (Fig. 3b), protein families associated with vesicle trafficking are also highly expressed. Gene families of the adaptin-, COP-, snare-, retromer and especially small GTPases show a significant expansion in transcription in comparison to the anaerobic parasite *G. intestinalis* or yeast (Fig. 3a). In comparison with that of humans, the expressed gene family sizes are comparable if not larger (except for the clathrin family; Fig. 3a), which is even more astonishing considering the lack of tissue-specific expression in *T. vaginalis*. This expansion underscores the importance of endocytic uptake of extracellular substrates in *T. vaginalis* and the combination of “sticky” N-terminal domains with a C-terminal domain recognized by the endocytic machinery may hint at trogocytosis similar to that observed in *Entamoeba* [17], or a rapid recycling of the plasma membrane and exchange of surface molecules to evade the human immune system as it occurs in trypanosomes [84].

Expression of candidate proteins increases the ability of T1 parasite cells to adhere to human tissue by at least 100% (Fig. 4a). In addition, the failure of the malic enzyme (TVAG_183790) to do the same refutes a potential moonlighting function of hydrogenosomal proteins in the parasite’s adhesion under the culture conditions used. This is in line with a series of papers that observed no moonlighting function for any of the proteins (including the malic enzyme) that otherwise only localize to the parasite’s hydrogenosomes [1, 13, 27, 85, 86].

Details of how the Pmp and BspA proteins are involved in adhesion and how they might subsequently be recycled remain obscure. Our sequence analysis shows that both members of the Pmp protein family analyzed possess endocytic motifs (Fig. 3c) and the data hint at a putative function in mediating host specificity. Expression of Pmp TVAG_140850 and BspA TVAG_240680 increases the ability of *Tet. gallinarum* (a pathogen naturally infecting birds) to adhere to human tissue (Fig. 4b). For chlamydial Pmps it was suggested that the specific FxxN and GGA[I/L/V] motifs are either directly or indirectly involved in mediating interactions with human receptors [41]. Based on this, Pmps might recognize specific host cell structures subsequently leading to the endocytosis of host material. The LRR domain of BspA proteins possesses putative functions in binding of host cell receptors and triggering a signaling cascade that promotes bacterial invasion [38]. *Trichomonas vaginalis* is, however, an extracellular parasite that secretes exosome-like vesicles, which play a role in the parasite’s attachment to host cells and modulate the human immune response [13]. Since the analyzed BspA protein TVAG_240680 (with a TMD and a 15 residues CCT without obvious signals for endocytosis) was found to be present in the pathogen’s exosome proteome, it might be associated with priming host cell tissue for parasite binding and eventual colonization as shown for *Tannerella forsythia*, *Treponema denticola* and other bacteria [36]. Similarly, some parasite BspA proteins could contribute to parasite binding to prokaryotes and other members of the microbiota to eventually mediate their phagocytosis [63, 64].

In prokaryotes, BspA and Pmp proteins aid attachment to host tissue. In *T. vaginalis*, this might be the case for those that carry a TMD and were found to be part of the pathogen’s surface proteome; it seems natural to assume that they are anchored into the plasma membrane of the eukaryotic parasite. It is evidently more complicated than that. The BspA and Pmp HA-fusion constructs localize to intracellular compartments and not the plasma membrane (Fig. 5a, Additional file 2: Figure S2, Additional files 4, 5, 6: Figures S4–S6). The defined localization around the entire nucleus is typical for the endoplasmic reticulum of the parasite that embraces the nucleus in several layers and is largely absent from the remaining cytosol, while the two adjacent rings at the apical end, and in close proximity to the nucleus, are typical for the Golgi apparatus of trichomonads [76, 77]. However, a sole localization of the analyzed BspA and Pmp proteins to compartments of the endomembrane system appears unlikely, as it is somewhat incompatible with the detection of some as part of a surface proteome analysis [34], the presence of the BspA protein TVAG_240680 in exosomes [13], and the increasing adhesion to host tissue we observed.

Despite these observations, an ER/Golgi localization for TVAG_166850 has been previously reported and agrees with its retention in the ER [78]. This targeting to endosomal compartments occurs independently of N-terminal signal peptides as none are detectable. It is possible that the majority of the protein resides in the ER and Golgi and is only transported to the surface in small concentrations. Alternatively, the missing membrane localization could be due to a cleavage-induced secretion of the protein, since the transmembrane domain of TVAG_166850 was shown to possess a cleavage site recognized by a specific membrane located rhomboid protease (*Tv*ROM1) [78]. Interestingly, for the Pmp and BspA protein, the N-terminal tag position leads to a localization shift to the lysosome and has a significant impact on its morphology (Fig. 6). Cultures expressing N-terminally tagged BspA and Pmp proteins show one lysosome, which is massively expanded. This is maybe due to the high number of incorporated proteins. The switch from a C- to a N-terminal tag also interferes with their inferred function leading to a significant decrease in the ability to mediate adhesion (Additional file 3: Figure S3). The latter underscores that an ER/Golgi localization is associated with their role in mediating adhesion. For the Pmp protein this is supported by former findings, where it has been shown that Pmp21 from *Chlamydia pneumoniae* is processed post-translational, leaving only the N-terminal part that acts as adhesin and therefore is essential for activity [44].

The localizations observed might overall complicate interpretations, but they are robust. Neither the use of different detergents nor GFP-tagging (and live-imaging) changed the localizations. Other proteins expected to be anchored into the plasma membrane have been observed to predominantly localize to the secretory system [78, 87], suggesting a more common mechanism is behind this in *Trichomonas*. Dedicated future investigations will be required to further our knowledge on the molecular cell biology of *Trichomonas* surface proteins mediating host–parasite interactions.

There is a wealth of diversity amongst both protein families in *T. vaginalis*. The parasite expresses many hundreds of BpsA genes (1.2% of which has been identified experimentally at the protein level on the cell surface) and at least two-dozen Pmp-encoding genes, all of which differ considerably in their primary sequence and even domain architecture. Only about 27% of the BspA, but roughly 90% of the Pmp proteins expressed, carry a TMD (Fig. 3a) that would allow them to be anchored into the plasma membrane to serve direct parasite adhesion. The same is true for the motifs recognized by the endocytic machinery; they are only infrequently found and rarely in combination with the other mentioned domains (Fig. 3c). It appears as if these two protein families are part of an ‘evolutionary playground’, with little selection pressure acting on a conserved set of domains simultaneously.

## Conclusions

Our results show that, although present in all trichomonad species analyzed, the massive expansion of the expression of genes belonging to both the Pmp and BspA protein family is restricted to *T. vaginalis*. This indicates that they play an important role for the infection of the human host. Furthermore, the common presence among the trichomonad species provides evidence for an ancient origin, possibly through HGT, which occurred prior to the early evolution of the parabasalids. The presence of several endocytic motifs within both protein families suggests that some of them play a role either in the endocytosis of host material or possibly the evasion of the host immune system by dynamically remodeling the surface proteome, both of which are essential components of the *T. vaginalis* infection. This is further underlined by increased expression levels of proteins associated with vesicle formation and intracellular trafficking. We demonstrated that the expression of specific BspA and Pmp proteins increased the adherence of the low-infective T1 strain of *T. vaginalis*, as well as the ability of the bird infecting *Tet. gallinarum* to bind to human host tissue, although the underlying mechanisms still need to be elicited. In contrast, the shared cell surface BspA and Pmp protein families across the investigated trichomonads and the demonstration that some family members are involved in binding to host cells, might have contributed to the zoonotic potential of some of these parasites, assuming that one or more family members bind to shared mucosal features across birds and mammals including humans [57]. In particular the bird-infecting *T. gallinae* closely related to the buccal *T. tenax* from mammals, and now known to be common among both pet mammals and humans, might be a particular case in point [57, 58]. What remains puzzling are the localizations that we and others (e.g. [78, 87]) observed. Together with the change in lysosome morphology, and expansion in proteins that orchestrate vesicle biology, this hints at unexplored avenues of *T. vaginalis* cell biology.

## Additional files

**Additional file 1: Figure S1.** Expression confirmation by reverse-transcriptase PCR.

**Additional file 2: Figure S2.** **a** Co-localization experiments were performed using LysoTracker (red) and 4′,6-diamidin-2-phenylindol (DAPI). **b** As a control, data of co-localization experiments are shown where no α-HA specific signal could be observed, excluding any bleedthrough from the LysoTracker channel. *Scale-bar*: 5 µm.

**Additional file 3: Figure S3.** Overexpression of the N-terminally tagged Pmp (TVAG_212570) and BspA (TVAG_240680) fusion proteins were tested with regard to their ability to increase the adherence of the *T. vaginalis* T1 wt strain. Top: examples for every assay are shown, each white or green dot represents one adhering parasite. Below the results of four independent experiments. T-tests were performed for the analysis of statistical significance compared to *T. vaginalis* wildtype strain T1 (***P < 0.0001, **P < 0.001, *P < 0.05).

**Additional file 4: Figure S4.** Expression of candidate proteins in *T. vaginalis* FMV1 strain. Candidate proteins were detected using a α-HA antibody (green) and the nuclei by 4′,6-diamidin-2-phenylindol (DAPI; blue). Compared to the T1 strain experiments, no change could be observed. White dashed lines highlight the periphery of the vaginal epithelial cells. *Scale-bar*: 5 µm.

**Additional file 5: Figure S5.** Localizations were further verified by using different detergents. The proteins were localized by a specific antibody against the HA tag (green), the nucleus was detected by using 4′,6-diamidin-2-phenylindol (DAPI; blue). Except for the N-terminal tagged Pmp (TVAG_212570) and BspA (TVAG_240680) protein, which were again found to reside inside a single enlarged lysosome, all the other constructs analyzed show the same ER/Golgi localization. *Scale-bar*: 5 µm.

**Additional file 6: Figure S6.** Candidate proteins were additionally detected using a C-terminal GFP tag (green) together with DAPI-staining (blue). Although only weak signals were detected, they verify our observations with the HA-tag and fixed cells. *Scale-bar*: 5 µm.

## Data Availability

The RNA-Seq datasets generated during the present study are publicly available through the National Center for Biotechnology Information (NCBI, https://www.ncbi.nlm.nih.gov) under the following accession numbers: SRX2052873 (*Pentatrichomonas hominis*), SRA318841 (*Tetratrichomonas gallinarum*), SRX2052874 (*Trichomitus batrachorum*), SRX2052872 (*Trichomonas gallinae*) and SRX2052871 (*Trichomonas tenax*).

## Abbreviations

BspA: Bacteroides surface protein A
Pmp: polymorphic membrane protein
ER: endoplasmic reticulum
AP2: adaptor protein 2
Rab: Ras-related proteins in brain
LRR: leucin-rich repeat
VEC: vaginal epithelial cells
FCS: fetal calf serum
ORF: open reading frame
BLAST: basic local alignment search tool
TMD: transmembrane domain
SCS: succinyl-CoA synthetase
RT: room temperature
DAPI: 4′,6-diamidin-2-phenylindol
HGT: horizontal gene transfer
CCT: C-terminal cytoplasmic tail
