# Supplementary figures and images for: Characterization of the BspA and Pmp protein family of trichomonads
Source: Parasit Vectors. 2019 Aug 19;12:406. doi: 10.1186/s13071-019-3660-z (PMC6701047; doi:10.1186/s13071-019-3660-z)

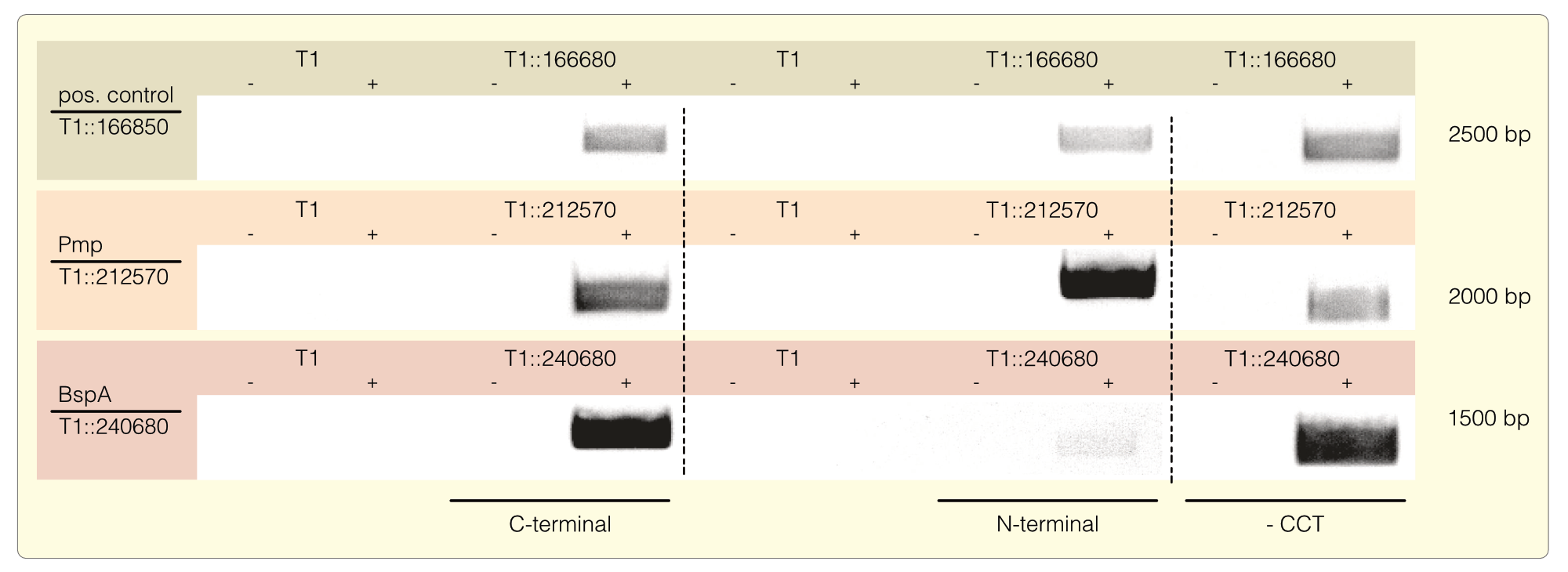

Supplement: Supplementary file 1 — Additional file 1: Figure S1. Expression confirmation by reverse-transcriptase PCR. [file 13071_2019_3660_MOESM1_ESM.tiff]

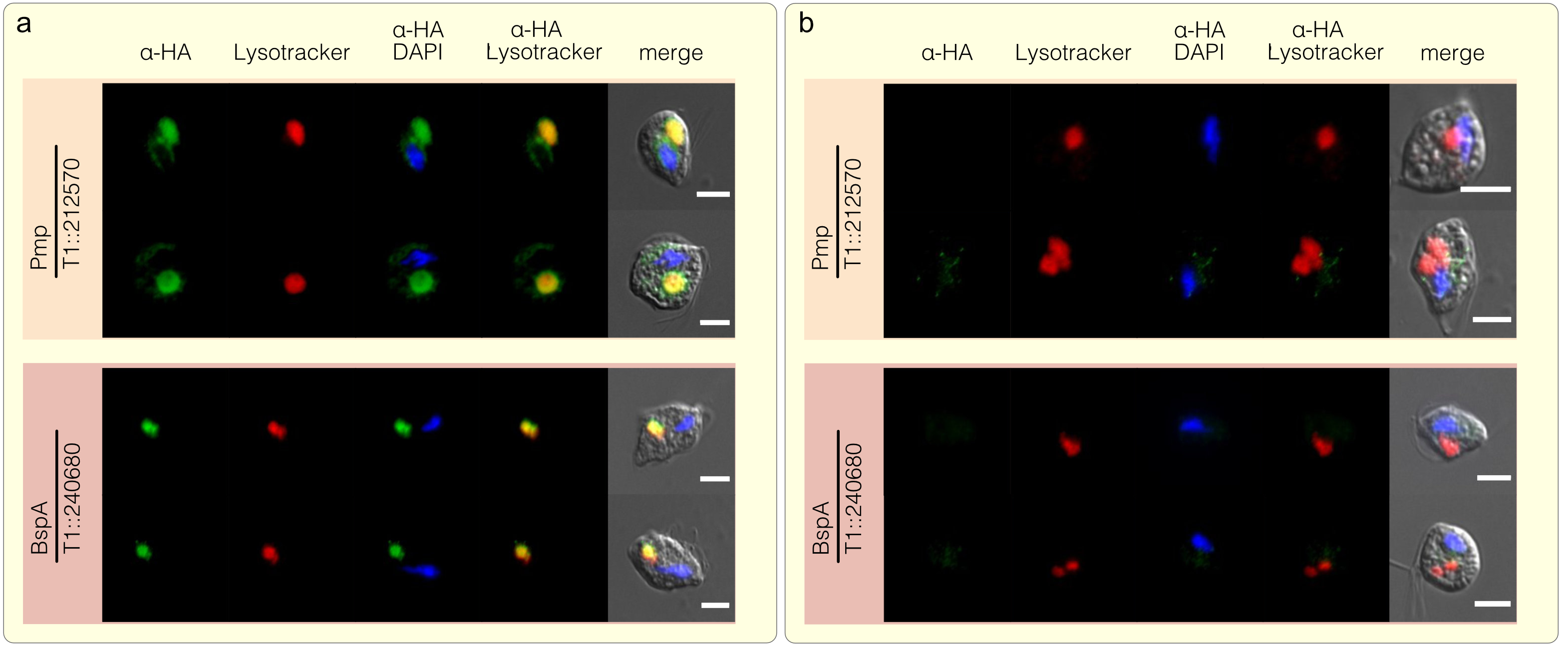

Supplement: Supplementary file 2 — Additional file 2: Figure S2. a Co-localization experiments were performed using LysoTracker (red) and 4′,6-diamidin-2-phenylindol (DAPI). b As a control, data of co-localization experiments are shown where no α-HA specific signal could be observed, excluding any bleedthrough from the LysoTracker channel. Scale-bar: 5 µm. [file 13071_2019_3660_MOESM2_ESM.tiff]

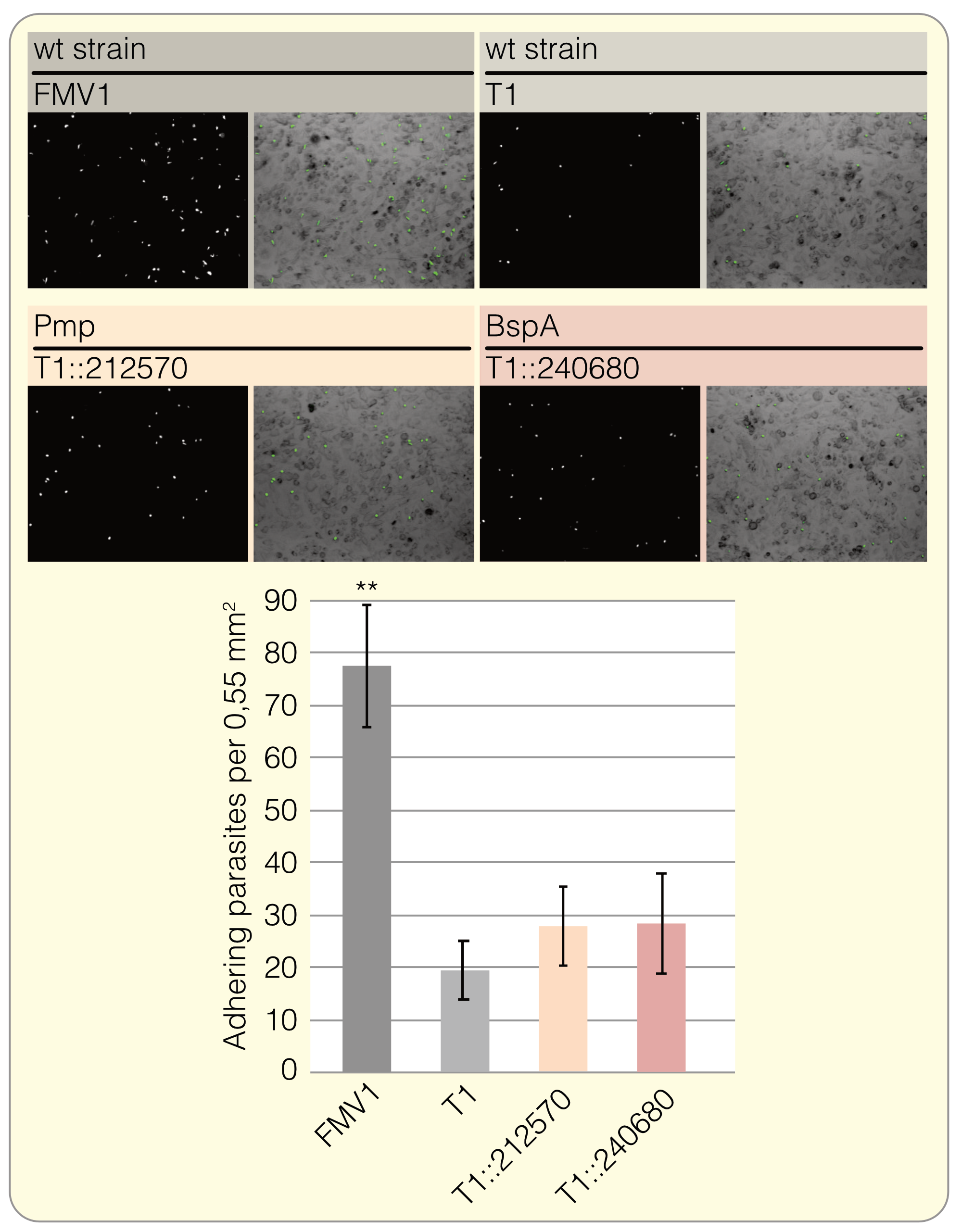

Supplement: Supplementary file 3 — Additional file 3: Figure S3. Overexpression of the N-terminally tagged Pmp (TVAG_212570) and BspA (TVAG_240680) fusion proteins were tested with regard to their ability to increase the adherence of the T. vaginalis T1 wt strain. Top: examples for every assay are shown, each white or green dot represents one adhering parasite. Below the results of four independent experiments. T-tests were performed for the analysis of statistical significance compared to T. vaginalis wildtype strain T1 (***P < 0.0001, **P < 0.001, *P < 0.05). [file 13071_2019_3660_MOESM3_ESM.tiff]

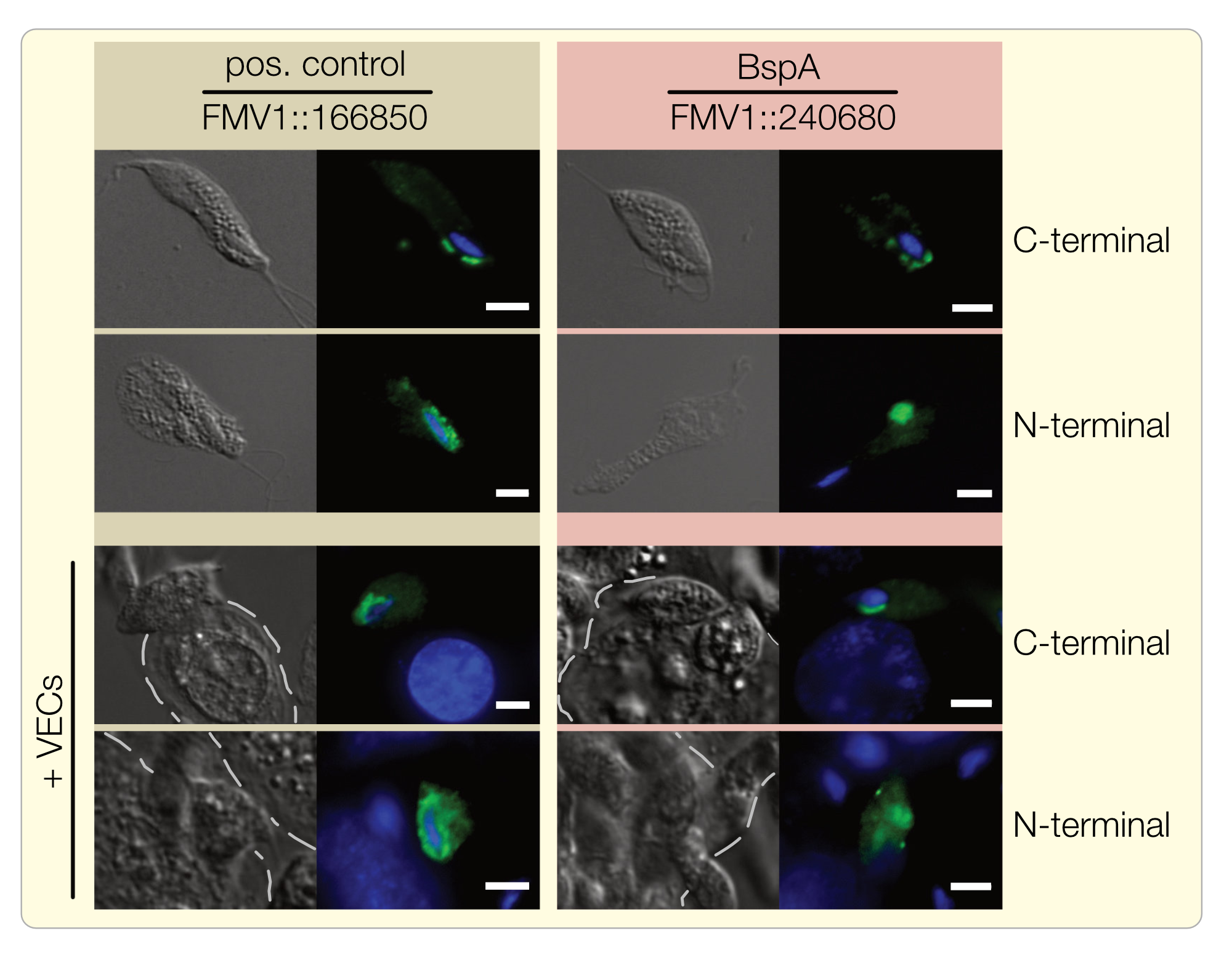

Supplement: Supplementary file 4 — Additional file 4: Figure S4. Expression of candidate proteins in T. vaginalis FMV1 strain. Candidate proteins were detected using a α-HA antibody (green) and the nuclei by 4′,6-diamidin-2-phenylindol (DAPI; blue). Compared to the T1 strain experiments, no change could be observed. White dashed lines highlight the periphery of the vaginal epithelial cells. Scale-bar: 5 µm. [file 13071_2019_3660_MOESM4_ESM.tiff]

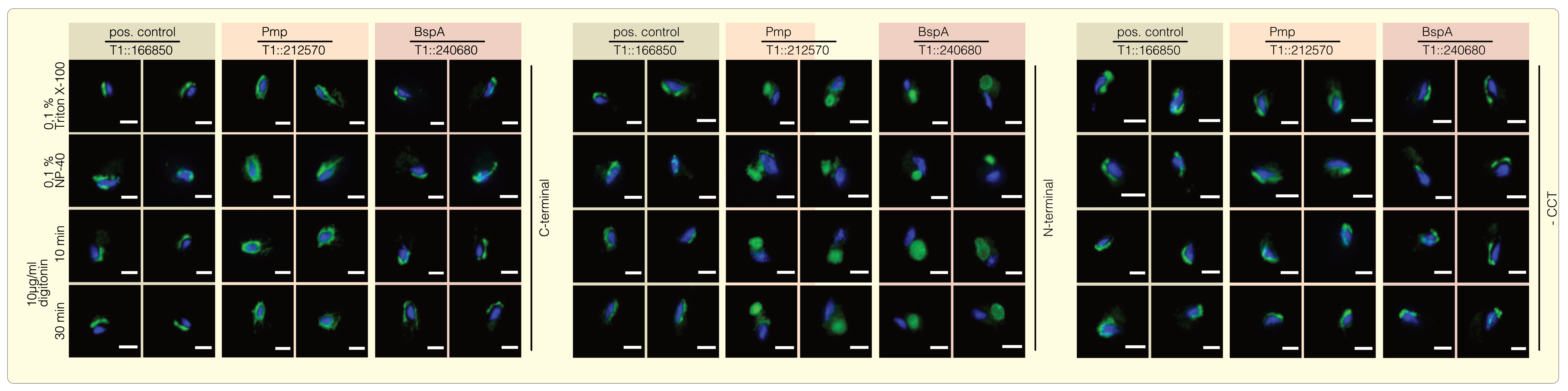

Supplement: Supplementary file 5 — Additional file 5: Figure S5. Localizations were further verified by using different detergents. The proteins were localized by a specific antibody against the HA tag (green), the nucleus was detected by using 4′,6-diamidin-2-phenylindol (DAPI; blue). Except for the N-terminal tagged Pmp (TVAG_212570) and BspA (TVAG_240680) protein, which were again found to reside inside a single enlarged lysosome, all the other constructs analyzed show the same ER/Golgi localization. Scale-bar: 5 µm. [file 13071_2019_3660_MOESM5_ESM.tiff]

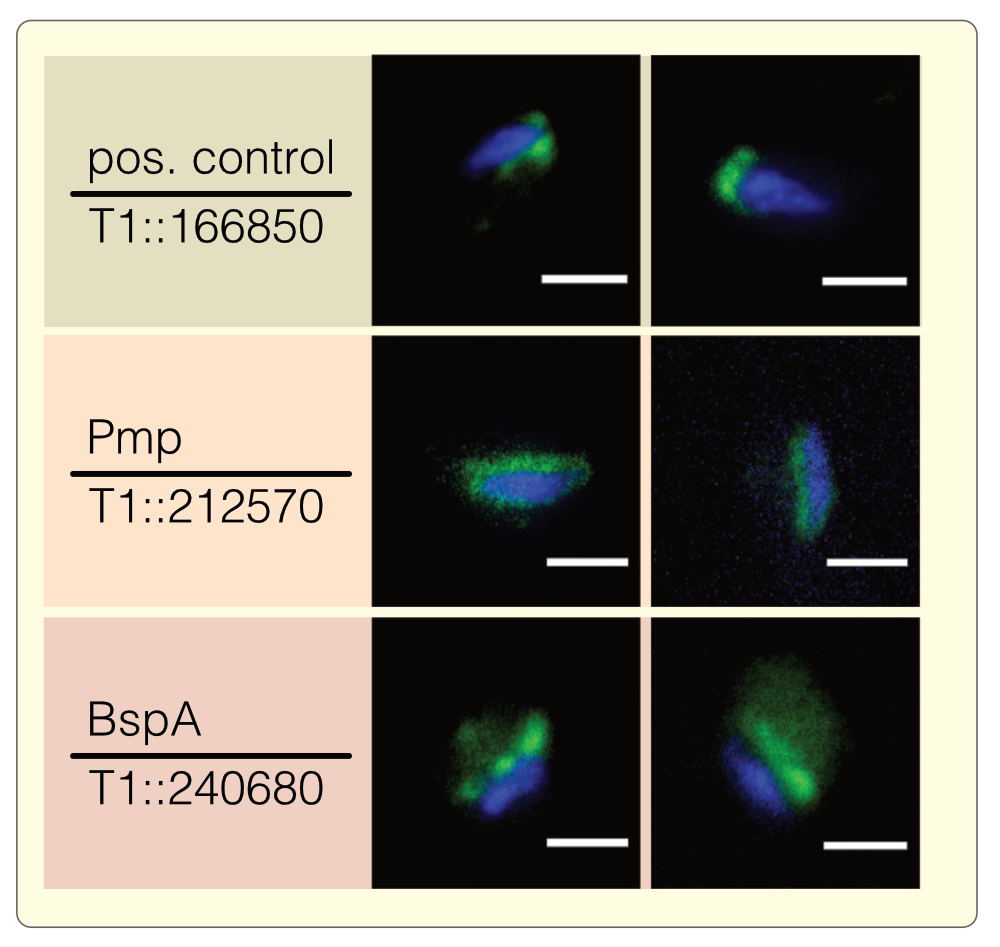

Supplement: Supplementary file 6 — Additional file 6: Figure S6. Candidate proteins were additionally detected using a C-terminal GFP tag (green) together with DAPI-staining (blue). Although only weak signals were detected, they verify our observations with the HA-tag and fixed cells. Scale-bar: 5 µm. [file 13071_2019_3660_MOESM6_ESM.tiff]
